# Optimizing Mineralocorticoid Receptor Antagonist Therapy in Heart Failure Through Integrated Care Strategies

**DOI:** 10.1016/j.jacadv.2026.102845

**Published:** 2026-06-17

**Authors:** Tina B. Marvasti, Kevin R. Murray, Gloria Ho, Holden Lowes, Stella Kozuszko, Margaret Brum, Natasha Verhoeff, Chun Po S. Fan, Jacob A. Udell, Steven Gregory Hershman, James L. Januzzi, Alexander T. Sandhu, Heather J. Ross, Michael A. McDonald, Juan G. Duero Posada, Yasbanoo Moayedi

**Affiliations:** a Ted Rogers Centre for Heart Research, Ajmera Transplant Centre, University of Toronto, Toronto, Canada; b Cardiology Division, Women’s College Hospital, University of Toronto, Toronto, Canada; c School of Information, University of Texas at Austin, Austin, Texas, USA; d Cardiology Division, Massachusetts General Hospital, Harvard Medical School, Baim Institute for Clinical Research, Boston, Massachusetts, USA; e University of California-Los Angeles, Los Angeles, California, USA

**Keywords:** guideline-directed medical therapy, heart failure, hyperkalemia, mineralocorticoid receptor antagonist, potassium binding

## Abstract

**Background:**

Hyperkalemia is a critical barrier to mineralocorticoid receptor antagonist (MRA) optimization in heart failure (HF) patients with reduced ejection fraction (HFrEF).

**Objectives:**

The objective of the study was to explore and evaluate a pragmatic, interdisciplinary strategy to safely optimize MRA use in high-risk patients.

**Methods:**

We conducted a prospective, observational study comparing patients at risk of hyperkalemia with HFrEF who were exposed to structured MRA titration, via dietitian-guided potassium restriction, remote monitoring, and potassium binder use, to a propensity-matched cohort followed on Medly, a telemonitoring program. A matched win-ratio was applied to assess the hierarchical composite outcome between the 2 cohorts. Outcomes evaluated were MRA optimization, left ventricular ejection fraction improvement, hyperkalemia events, and HF-related hospitalizations.

**Results:**

Among 185 patients (51 exposure and 134 Medly), the exposure group had a higher baseline potassium (5.1 vs 4.2 mmol/L, *P* < 0.001) and a higher use of potassium binders compared to the Medly group (53% vs 7%, *P* < 0.001). After 1:1 propensity matching (82 pairs), the exposure cohort had significantly higher MRA optimization rates (HR: 4.67; 95% CI: 1.93-11.27; *P* < 0.001), and fewer HF hospitalizations (*P* = 0.014). The composite win ratio which included death, HF hospitalization, left ventricular ejection fraction improvement, and hyperkalemic events significantly favored the exposure group (2.5; 95% CI: 1.3-6.35; *P* = 0.005).

**Conclusions:**

A multidisciplinary approach combining dietary counseling, remote monitoring, and potassium binders was associated with improved optimization of MRA therapy in HFrEF patients at risk of hyperkalemia. This model may offer potential clinical benefit and underscores the value of team-based care in HF management.

Heart failure (HF) is a growing health issue with high rates of mortality and morbidity, costing over $30 billion annually in the United States due to more than 1 million hospitalizations each year.^1^ Evidence indicates that guideline-directed medical therapy (GDMT) results in significant mortality and morbidity benefits in patients with HF with reduced ejection fraction (HFrEF) which constitute approximately half of all HF diagnoses.^2^ GDMT encompasses evidence based doses of 4 main therapies including beta-blockers, mineralocorticoid receptor antagonists (MRAs), sodium glucose cotransporter-2 inhibitors (SGLT2i) and a renin-angiotensin inhibitor; this last class includes angiotensin converting enzyme inhibitors (ACEI), angiotensin receptor blockers (ARBs) and angiotensin receptor neprilysin inhibitors (ARNIs) with the latter therapy being preferred. Despite the clear mortality benefits of GDMT, its implementation in clinical practice remains suboptimal.^3^^,^^4^

Among these treatments, MRAs present unique challenges for initiation and dose adjustment due to the risk of hyperkalemia.^5^ This medication class inhibits the binding of aldosterone to mineralocorticoid receptors promoting sodium and fluid excretion while increasing potassium reabsorption. At the level of cardiomyocytes, steroidal MRA medications such as spironolactone and eplerenone have also been shown to prevent myocardial fibrosis and help with extracellular matrix turnover in HF.^6^ Treatment of HFrEF with MRA results in lower rates of major adverse events including death or hospitalization.^7^ Based on this evidence, MRAs are one of the main 4 pillars recommended for HFrEF therapy as per American, European, and Canadian cardiovascular society guidelines.^3^^,^^8^^,^^9^

Despite strong recommendations, only one-third of eligible patients receive an MRA, with hyperkalemia, a potentially serious side effect associated with arrhythmias, hospitalization, and death, being the most frequently cited barrier.^5^^,^^10^^,^^11^ Recently, even with the selective nonsteroidal MRA finerenone, structured potassium monitoring and dose adjustments were necessary to minimize hyperkalemia.^12^ To support MRA use in patients at risk of hyperkalemia, potassium binding medications have been explored for their ability to maintain normal potassium levels despite use of MRA. In the DIAMOND (Patiromer for the Management of Hyperkalemia in Subjects Receiving RAASi Medications for the Treatment of Heart Failure) trial, those randomized to potassium binding had a reduced hyperkalemia risk and a decreased need for MRA dose reduction compared to the control group.^13^

In this study, we explored a streamlined approach combining professional dietary counseling, remote monitoring, and the use of a potassium binder for MRA titration. We hypothesized that a structured interdisciplinary approach could help optimize MRA therapy, which could ultimately lead to reduced hyperkalemic events.

## Methods

### Study design and participants

This study is reported according to the cohort study checklist for Strengthening the Reporting of Observational Studies in Epidemiology (STROBE). Institutional regulatory approval was obtained (QI-21-0253 with consent waived for prospective study and REB 23-5150). In this observational, single-center study, we prospectively enrolled patients undergoing interdisciplinary MRA titration and compared them to a propensity-matched cohort of patients with HFrEF followed on Medly, a clinically validated outpatient HF telemonitoring program.^14^

The exposure group of this study included consecutive patients with HFrEF at risk for hyperkalemia (potassium >4.9 mmol/L at the time of study enrollment) and either on a suboptimal dose of MRA or without MRA therapy. Patients were onboarded on our Medly program and received dietitian assessment within 1 to 2 weeks of referral.^14^ As part of the dietary counselling, patients kept a 3-day log of their meal intake using a paper diary to review in person or on the phone with our dietitian. The dietitian provided tailored counseling on dietary modifications to minimize potassium intake, while also addressing sodium and fluid restrictions. Each patient was reviewed in weekly interdisciplinary rounds. Patients with advanced HF therapies (ie mechanical devices or post cardiac transplant), a documented intolerance (ie allergic reaction) to MRA therapy as well as those with eGFR <30 mL/kg/1.73 m^2^ or on dialysis were excluded from the study. Baseline demographics, potassium level, left ventricular ejection fraction (LVEF), and MRA dose were documented on a REDCap platform.^15^ The Medly cohort, the comparator, consisted of patients onboarded in the same contemporaneous period. Patients on Medly are provided with a blood pressure cuff, a scale, and a phone and are assigned to a nurse-coordinator for alerts and potential uptitration of GDMT.^14^

Both cohorts were followed for 1 year. The primary outcome was MRA dose optimization, whereas secondary outcomes included incidence of hyperkalemic events, changes in LVEF, and HF-related hospitalization and mortality. Finally, Medly adherence was defined by the number of days where vitals were entered over 1-year period.

### Statistical analysis

Clinical characteristics were summarized using descriptive statistics. Continuous variables were characterized using median and first/third quartiles; dichotomous or polytomous variables were characterized using frequencies. Between-group differences in continuous and categorical baseline variables were assessed using Wilcoxon rank-sum and Fisher exact tests, respectively. Propensity scores for each patient were estimated using a flexible boosting method. The following clinical characteristics at the time of onboarding (baseline) were considered in PS: age, sex, body mass index (BMI), MRA, ARNI usage, LVEF, implantable cardioverter defibrillator implant, B-type natriuretic peptide (BNP), MRA dose at baseline, and prior hyperkalemic events. Exposure patients were matched 1:1 with Medly patients based on PS. A total of 82 patients (41 matched-pairs) were included.

Postmatching covariate imbalance was assessed and summarized using standardized differences. The Kaplan-Meier (KM) survival method was used to estimate postmatching survival rates, and the stratified log-rank test was used to compare the survival curves between treatment groups. Cumulative incidence rates were estimated for hospitalization due to HF, hyperkalemic events, and MRA target dose tolerance using the Gray subdistribution method, with death as a competing risk. Between-cohort differences in the cumulative incidence rates were evaluated using stratified Gray tests. The effect of exposure was also quantified using a stratified Cox proportional hazard model for mortality, and stratified Fine and Gray subdistribution hazard models for the aforementioned outcomes with competing risks. Estimation of effect size through regression analysis was not feasible for HF hospitalization due to the absence of events in the exposure group. As a result, we have retained the analysis of this outcome using a KM curve and stratified log-rank test.

In addition, a matched win-ratio was applied to assess the hierarchical composite outcome of death, HF-related hospitalization at 1 year, LVEF improvement, and hyperkalemic events between the 2 cohorts.^16^ We defined LVEF improvement as an increase of more than 10% within the 1-year follow-up period.

## Results

### Study cohort and patient characteristics

Overall, 185 patients were included in the study, consisting of 51 patients enrolled in the exposure cohort from January 1, 2022 to May 2023 and 134 patients in the Medly observational cohort onboarded in the same period. The median age across both cohorts was 58.8 (IQR: 51-65.1) years, with the majority being males (77%) with a primary diagnosis of dilated cardiomyopathy (55%). Forty percent of patients had an implantable cardiac defibrillator with a baseline LVEF 30% (IQR: 22%-36%) and a BNP level of 167 (75-400) ng/L. Before matching, hyperkalemic events that restricted MRA titration were significantly more common in the exposure group with 35 (69%) affected, compared to 14 (10%) (*P* < 0.001). The exposure group had a higher baseline potassium (5.1 mmol/L) vs the control group (4.2 mmol/L; *P* < 0.001). Patients in the Medly group were younger (56.9 vs 62.8 years old; *P* < 0.001) and had a higher BMI (28 vs 24 kg/m^2^; *P* = 0.013) as shown in Table 1.

**Table 1:** Baseline Patient Characteristics in Unmatched Cohort

	Control (n = 134)	Exposure (n = 51)	*P* Value
Age (y)	56.9 (47.4-64.2)	62.8 (58.4-74.4)	<0.001
Male	101 (75%)	42 (82%)	0.43
Baseline BMI (kg/m^2^)	28.1 (24.7-31.1)	24.2 (22.1-29.5)	0.013
Prior hyperkalemic events	14 (10%)	35 (69%)	<0.001
HFrEF etiology			
Ischemic cardiomyopathy	35 (26%)	25 (49%)	
Dilated cardiomyopathy	80 (60%)	21 (41%)	
Restrictive cardiomyopathy	2 (1%)	0 (0%)	
Hypertrophic cardiomyopathy	4 (3%)	0 (0%)	
Other	13 (10%)	4 (8%)	
Baseline LVEF (%)	30.0 (22.0-35.0)	30 (23-37)	0.48
HF therapies			
Beta-blocker	133 (99%)	51 (100%)	1.00
ACE inhibitor/ARB/ARNI	134 (100%)	47 (92%)	0.005
ARNI	66 (49%)	23 (49%)	1.00
MRA	108 (81%)	33 (65%)	0.033
MRA (on target dose)	80 (60%)	13 (25%)	<0.001
SGLT2 inhibitor	121 (90%)	45 (88%)	0.79
Potassium binder	0 (0%)	2 (4%)	0.075
Furosemide	77 (57%)	23 (45%)	0.141
Baseline serum biomarkers			
Potassium level, mmol/L	4.2 (3.8-4.5)	5.1 (4.9-5.4)	<0.001
Sodium level, mmol/L	139 (137-141)	139 (137-142)	0.45
Creatinine level, mmol/L	100 (82-130)	102 (90.5-121.0)	0.78
BNP level, pg/mL	163 (68.8-389.0)	191 (102.0-403.0)	0.58

ACE = angiotensin-converting enzyme inhibitor; ARB = angiotensin receptor blocker; ARNI = angiotensin receptor-neprilysin inhibitor; BMI = body mass index; BNP = B-type natriuretic peptide; HF = heart failure; HFrEF = heart failure with reduced ejection fraction; LVEF = left ventricular ejection fraction (%); MRA = mineralocorticoid receptor antagonist; SGLT2 = sodium-glucose cotransporter-2.

With regards to baseline medications, both groups were similar with an overall 99% on a beta-blocker, 49% on ARNi, 90% were on SGLT2i, and fewer patients in the exposure cohort were on MRA therapy compared to the Medly cohort (65% vs 81%, *P* = 0.03). None of the patients in the observational cohort were on a potassium binder at baseline, whereas 2 patients (3.9%) in the exposure arm were already on a binder before the exposure. Follow-up to 1 year was complete for all patients with the exception of 8 patients who died during the follow-up.

### Outcomes before matching

Overall, only 4 patients (3%) received professional dietary counseling in the observational cohort as compared to all patients in the exposure group. Patients in the exposure arm had a significantly lower potassium level after dietitian counseling (4.7 vs 5.1 mmol). At 1-year follow-up, the exposure group had a similar higher rate of MRA optimization compared to the Medly cohort (94% vs 88%; *P* = 0.29) without a corresponding increase in hyperkalemic events (10% vs 12%; *P* = 0.80) or mortality (6% vs 5%; *P* = 1.00) before matching (Table 2). There was a commensurate higher use of potassium binders in the exposure group compared to the Medly group (53% vs 7%; *P* < 0.001). There was no difference in the use of Medly adherence (85% vs 77.4%; *P* = 0.13). The exposure group experienced a significant reduction in HF-related hospitalization (13% vs 0%; *P* = 0.004) and a significant improvement in LVEF (32% vs 37%; *P* = 0.009) (Table 2).

**Table 2:** Patient Outcome Characteristics on Follow-up in Unmatched Cohort

	Control (n = 134)	Exposure (n = 51)	*P* Value
Follow up MRA (optimized dose)	118 (88%)	48 (94%)	0.29
Episodes of hyperkalemic events	16 (12%)	5 (10%)	0.80
Death during study period	7 (5%)	3 (6%)	1.00
HF-related hospitalization	17 (13%)	0 (0%)	0.004
Dietitian referrals	4 (3%)	51 (100%)	<0.001
Follow up LVEF (%)	32 (24-40)	37 (28-48)	0.009
Rate of Medly adherence (%)	74.7 (37.07-95.98)	85.03 (66.60-95.63)	0.129

Abbreviations as in Table 1.

### Propensity score matching and win ratio and composite outcome analysis

**Figure 1:**
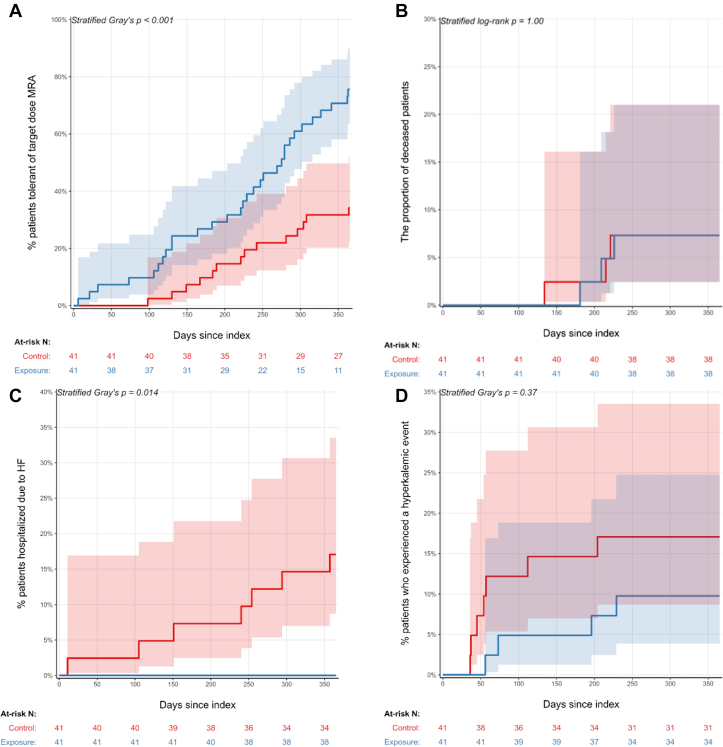
Outcomes Stratified by Exposure and Control Outcomes stratified by exposure and Medly (control) for the matched cohort as follows: (A) cumulative incidence curve for target MRA dose, (B) proportion of deceased patients, (C) hospitalization due to HF and (D) hyperkalemic events. HF = heart failure; MRA = mineralocorticoid receptor antagonist.

Patients in the exposure group were matched to the Medly group based on a propensity score. A total of 82 patients were included in the matched pair analysis. The quality of the matching was summarized by the distribution of the scores before and after matching, stratified by exposure group (Supplemental Figure 1). Among 41 matched pairs, there were 6 deaths, 4 hospitalizations, 20 patients with an LVEF improvement, and 4 hyperkalemic events. When compared to Medly in the KM estimates, the exposure cohort achieved a significantly higher target dose of MRA (HR: 4.67; 95% CI: 1.93-11.27; *P* < 0.001), a significantly lower HF-hospitalization rate (*P* = 0.014) and no difference in hyperkalemic event (HR: 0.57; 95% CI: 0.17-1.95; *P* = 0.37) or death (HR: 1.00; 95% CI: 0.20-4.96; *P* = 1.00) as shown in Figure 1. The win ratio for the composite outcome was 2.5 (95% CI: 1.3, 6.35; *P* = 0.005) in favor of the exposure group (Table 3).

**Table 3:** Win Ratio Analysis for the Composite Outcome Among the Matched Cohort

Outcome	# Of Wins for the Exposure Group	# Of Losses for the Exposure Group	Ties
Death	4	3	
Hospitalization	4	0	
Left ventricular ejection fraction improvement	15	5	
Hyperkalemic event	3	1	
Total	26	9	6

Win Ratio	95% CL	*P* Value
2.889	1.49-7.9	0.001

CL = confidence level

## Discussion

Concerns related to hyperkalemia are frequently cited as the primary barrier to prescribing or optimizing MRA therapy in patients with HFrEF, despite the well-documented benefits in reducing mortality and hospitalizations.^17^ This real-world study found that a structured, interdisciplinary approach of combining dietary counseling, remote monitoring and potassium binders was associated with significantly improved MRA optimization without increasing hyperkalemic events (Central Illustration). In patients with potassium-limiting MRA rates, the exposure group saw higher MRA rates with lower hyperkalemic events as compared to a propensity-matched and closely monitored Medly group.

**Central Illustration:**
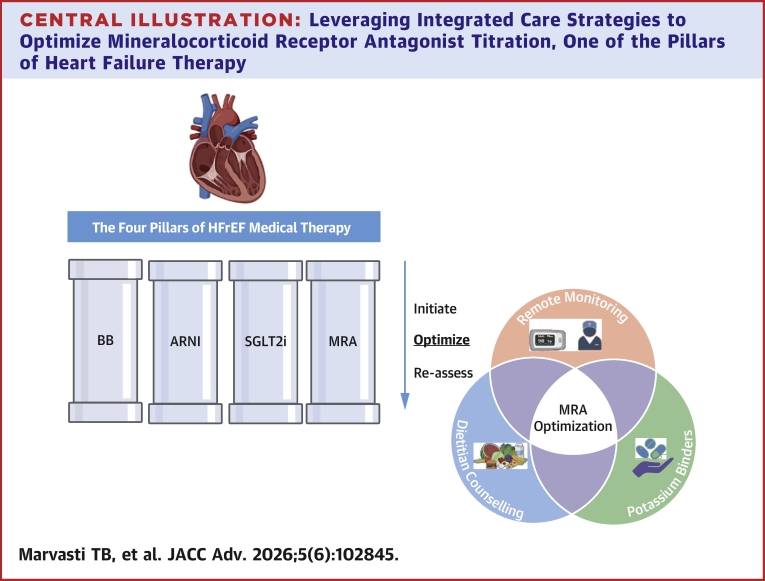
Leveraging Integrated Care Strategies to Optimize Mineralocorticoid Receptor Antagonist Titration, One of the Pillars of Heart Failure Therapy Combining multidisciplinary dietary counseling, remote monitoring, and potassium binders was associated with improved optimization of MRA therapy in HFrEF patients at risk of hyperkalemia. ARNI = angiotensin receptor-neprilysin inhibitor; BB = beta-blocker; HFrEF = heart failure with reduced ejection fraction; MRA = mineralocorticoid receptor antagonist; SGLT2i = sodium-glucose cotransporter-2 inhibitor.

Implementation of GDMT in real-world practice remains suboptimal^18^ with persistent gaps in the use of GDMT, with <30% of patients on optimal MRA therapy.^17^^,^^19^ In a recent systematic review, interdisciplinary titration clinics were associated with the most effective means of getting patients to target doses of GDMT.^18^ Patients in both arms of the study are followed by the Medly telemonitoring system in a quaternary setting which provides close monitoring and opportunities for GDMT uptitration. While our data shows exceptional adherence of GDMT in our Medly group with 99% on a beta blocker, 100% ACEI/ARB or ARNi and 81% on MRA, 90% on SGLT2i, there is a clear opportunity to improve rates of MRAs in patients who are at high risk of hyperkalemia. These findings suggest that targeted strategies addressing potassium management may provide incremental benefits beyond standard telemonitoring alone.

Our experience underscores the critical role of dietitian assessments in safely managing potassium levels. The dietary guidance provided was associated with significantly reduce baseline potassium in the majority of patients to a range where titration of MRA could occur doing so without the need for potassium binding therapies. These results are reminiscent of those from the DIAMOND Trial, where a substantial percentage of study participants with a history of hyperkalemia from MRA therapy did not experience it again when subsequently challenged. As nutritional sources of potassium are common, dietitian support may be invaluable, offering tailored guidance that empowers patients to achieve potassium control.

For patients whose potassium levels remained elevated (>4.9 mmol/L) despite dietary interventions, potassium binders were introduced. Medications like patiromer and sodium zirconium cyclosilicate represent an emerging class that reduce potassium levels by binding to potassium in the gastrointestinal tract to prevent absorption. A meta-analysis of 6 RCTs found that potassium binders improved MRA optimization without any significant risk to adverse events.^20^ In our exposure group, we found that only 50% of patients required a potassium binder to reach a target dose of 25 mg of spironolactone. While there is ongoing debate regarding the cost-effectiveness of potassium binders, in patients who have received dietitian-counseling, these agents can enable adequate MRA dosing, potentially contributing to reduced mortality, fewer HF hospitalizations and improved LVEF even when compared to closely monitored Medly cohort. While these agents may facilitate optimization, their cost, access, and patient burden are important considerations that may limit scalability across healthcare systems.

Our study has several limitations that may limit the generalizability of our findings. Notably, none of the patients in our exposure or Medly cohort were on finerenone, a selective non-steroidal MRA which may be associated with lower rates of hyperkalemia. While recent results from the CONFIDENCE trial suggest that the initiation of simultaneous SGLT2i and nonsteroidal MRA in patients with chronic kidney disease reduces the frequency of hyperkalemic events by 15 to 20%, hyperkalemia-related discontinuation still occurred despite strict exclusions, intensive laboratory monitoring and trial condition.^21^ Importantly, participants in this cohort were required to be on an ACEI/ARB rather than ARNI. Moreover, finerenone is not yet approved for use to treat HFrEF. Several additional limitations warrant consideration. First, the relatively small sample size, particularly in the context of a complex statistical framework including propensity matching and win-ratio analysis, limits the precision of effect estimates and increases the risk of residual confounding. We were unable to adequately match patients for baseline BMI and age. Consequently, our exposure cohort remains significantly older with lower BMI introducing potential confounders that our analysis may not fully capture. Furthermore, there were baseline differences between groups, including higher use of target-dose MRA therapy in the control group and a higher prevalence of prior hyperkalemic events in the exposure group, these imbalances would be expected to bias toward improved outcomes in the control arm, potentially attenuating the observed treatment effect despite matching. Lastly, several covariates may vary over time postindex; however, we lacked the longitudinal data needed to assess any time-dependent biases. These time-dependent variables (ie, medication titration, LVEF, BNP) were measured and incorporated at baseline only.

The exposure was inherently heterogeneous, incorporating dietary counseling, remote monitoring, and potassium binders, and the relative contribution of each component cannot be definitively determined. While our study primarily focused on MRA uptitration, optimizing potassium can also facilitate improved titration of ARNi/ACEI/ARB therapies. The improvement in LVEF observed in the exposure group compared to Medly is likely attributable to this combined effect. Additionally, the beneficial effects of dietary counseling may extend beyond potassium intake, encompassing broader nutritional guidance and support.

Finally, access to dietitians for potassium counseling is limited and interdisciplinary care on this level may not be scalable for the millions of HF patients. However, many available technologies and applications could offer complementary support for patients who lack dietitian access.

In conclusion, in this study we demonstrated that in a cohort of HFrEF patients followed by HF cardiologists at a quaternary cardiac care centre at risk of hyperkalemia, a streamlined interdisciplinary approach to dietary counselling, remote monitoring and potassium binders is a feasible strategy associated with improved initiation and titration of MRA therapy. This approach aligned with more wins in the comparison of death, HF related hospitalization and improvement in LVEF vs Medly patients without increasing the risk of hyperkalemic events. Such findings highlight the potential of targeted, team-based care models in achieving optimal GDMT in HF patients, particularly when dietitian support is available. Scaling this model could lead to improved outcomes, though further studies are needed to assess cost-effectiveness and wider accessibility of these resources in diverse health care settings.

Perspectives **COMPETENCY IN PATIENT CARE:** Effective optimization of MRA therapy in high-risk HFrEF patients requires close laboratory surveillance, individualized dietary counseling, and timely adjustment of therapy. Incorporating remote monitoring platforms and interdisciplinary care pathways may improve patient safety while enabling greater adherence to evidence-based HF therapies. Clinicians should recognize the importance of proactive hyperkalemia management to avoid premature discontinuation or underdosing of MRAs.

**TRANSLATIONAL OUTLOOK:** Future randomized studies are needed to determine whether multidisciplinary hyperkalemia mitigation strategies translate into long-term reductions in mortality and HF hospitalization. Additional investigation is also warranted to identify optimal approaches for integrating potassium binders, telemonitoring, and dietary interventions into routine HF care pathways. Expanding these models across diverse healthcare systems may further improve implementation of guideline-directed medical therapy in HFrEF.

## Funding support and author disclosures

This study received unrestricted funding by Otsuka Canada Pharmaceuticals Inc. The authors have reported that they have no relationships relevant to the contents of this paper to disclose.
